# Genetic diversity and geographical distribution of *Trypanosoma cruzi* DTUs in Mexico: A Systematic Review

**DOI:** 10.1590/0037-8682-0126-2026

**Published:** 2026-08-03

**Authors:** Raúl Edgardo Cruz-Cadena, Doireyner Daniel Velázquez-Ramírez, José Antonio De Fuentes-Vicente, Refugio Cruz-Trujillo, Josué Vidal Espinosa-Juárez, Abumalé Cruz-Salomón, Dulce Concepción Domínguez-Cruz

**Affiliations:** 1 Universidad Autónoma de Chiapas, Escuela de Ciencias Químicas, Ocozocoautla de Espinosa, Chiapas, México.; 2 El Colegio de la Frontera Sur, Departamento de Salud, San Cristóbal de las Casas, Chiapas, México.; 3 Universidad para el Bienestar Benito Juárez García, Carrera de Medicina Integral, San Andrés Larráinzar, Chiapas, México.; 4 Universidad Autónoma de Ciencias y Artes de Chiapas, Instituto de Ciencias Biológicas, Tuxtla Gutiérrez, Chiapas, México.; 5 Universidad Pablo Guardado Chávez, Departamento de Químicos Farmacobiólogos, Tuxtla Gutiérrez, Chiapas, México.

**Keywords:** American trypanosomiasis, Genotype, Zoonoses, Triatominae

## Abstract

*Trypanosoma cruzi* exhibits substantial genetic diversity, classified into discrete typing units (DTUs) that may differ in geographical distribution, host associations, and epidemiological relevance. Although several DTUs have been reported in Mexico, evidence on their national distribution across vectors, reservoirs, and humans remains fragmented. A systematic review was conducted following PRISMA guidelines to synthesize available information on the geographical distribution, host associations, and epidemiological cycles of *Trypanosoma cruzi* DTUs in Mexico. Seven scientific databases were searched for studies published between January 2006 and July 2025. Inclusion criteria focused on studies reporting molecular identification of *T. cruzi* DTUs in vectors, reservoirs, and humans within Mexican territory. Forty-seven studies were analyzed, providing a total of 1,337 individual records. The analysis confirmed the circulation of six DTUs (TcI-TcVI) in 22 states, with TcBat being absent from the records. TcI was identified as the most frequent and widely distributed lineage, present in 20 of the 22 states. Veracruz documented the highest genetic diversity, with all six DTUs reported. The genus *Triatoma* and domestic dogs exhibited the highest diversity of reported DTUs, acting as key epidemiological bridges between transmission cycles. TcI is the predominant lineage in Mexico, demonstrating high connectivity across all transmission cycles. This systematic review highlights significant information gaps in northern Mexico and underscores the need for strengthened surveillance to better understand the impact of *T. cruzi* genetic diversity on public health.

## INTRODUCTION

Chagas disease (CD), caused by the protozoan parasite *Trypanosoma cruzi*, is a significant parasitic infection in Latin America. Due to migration and globalization, it has become a global public health concern^1^. Transmission primarily occurs through contact with infected feces of hematophagous triatomine insects. However, other routes exist, such as blood transfusion, congenital transmission, or oral contamination^2^. 

Mexico is a key endemic region, with CD widely distributed, especially in central and southern states where ecological and socioeconomic conditions favor vector persistence^3^ ^,^ ^4^. Estimates of *T. cruzi* infection in Mexico range from 1.2 million^5^ to 4.06 million individuals^6^. This variability likely reflects underdiagnosis, limited national surveillance, and suboptimal sensitivity of routine diagnostic tools^7^ ^,^ ^8^. These factors highlight the need to strengthen epidemiological monitoring across all transmission cycle components: vectors, reservoirs, and humans.

*T. cruzi* is known for its remarkable genetic heterogeneity, classified into seven discrete typing units (DTUs). Before this formal consensus, researchers classified the parasite using zymodemes or early lineages. These historical classifications map accurately to modern DTUs, allowing the integration of earlier studies into current analyses. These units are designated as TcI through TcVI, and TcBat^9^ ^,^ ^10^. These DTUs are linked to distinct epidemiological transmission cycles and variations in clinical presentation^11^ ^-^ ^13^. The coexistence of multiple DTUs within the same geographic region adds complexity, challenging simplistic models of transmission and disease manifestation.

Previous reviews show a wide distribution of DTUs in the Americas, with TcI being the most frequent^14^ ^-^ ^16^. In Mexico, six DTUs (TcI-TcVI) have been documented. However, available evidence is fragmented, mainly from continental reviews or isolated regional studies^14^ ^,^ ^17^ ^-^ ^31^, limiting a comprehensive understanding of national patterns. No systematic review has focused exclusively on Mexico, integrating vectors, reservoir hosts, and human infections. This gap is significant given Mexico's ecological diversity and the documented co-occurrence of multiple DTUs in shared transmission settings^32^.

This systematic review compiles and analyzes evidence published between January 2006 and July 2025 to provide an updated, integrated overview of *T. cruzi* DTU distribution in Mexico. The analysis encompasses vectors, reservoir hosts, and human cases, offering a comprehensive national perspective. Prior studies offer continental overviews or regional analyses within Mexico^14^ ^,^ ^15^ ^,^ ^32^ ^-^ ^34^. This research is the first systematic review focused solely on Mexico, integrating molecular data from all components of the transmission cycle. By synthesizing twenty years of evidence, we aim to strengthen surveillance and inform evidence-based policies in Mexico.

## METHODS

Following the Preferred Reporting Items for Systematic Reviews and Meta-Analyses (PRISMA 2020) guidelines^35^, an exhaustive search was performed across seven scientific platforms: Public/Publisher MEDLINE (PubMed), ScienceDirect, Web of Science, the Network of Scientific Journals from Latin America and the Caribbean, Spain and Portugal (Redalyc), the Scientific Electronic Library Online (SciELO), Dialnet, and the Directory of Open Access Journals (DOAJ), covering studies published from 2006 to July 16, 2025.

### Inclusion and Exclusion Criteria

The review included only those studies reporting the identification of *T. cruzi* DTUs in Mexico, as well as works involving molecular analysis, genotyping, or studies of reservoirs, vectors, and humans where the specific DTU was determined. Studies were excluded if they:

- Did not provide information regarding Mexican territory.
- Only reported the presence of samples positive for *T. cruzi* without DTU identification or characterization.
- Were based solely on serological tests without genotypic or molecular analysis.
- Contained insufficient information or lacked clear data on the DTU (Supplementary Figure S1).

### Search Strategy

The search strategy combined keywords related to the etiological agent, the DTUs, the hosts, and the geographical delimitation. To maximize search sensitivity and exhaustiveness, terms were used in both English and Spanish, combined using Boolean operators (AND, OR). The strategy was structured around two main search strings, which served as the basis for generating various combinations:

- Search String 1: ("*Trypanosoma cruzi*" OR "Chagas disease") AND ("discrete typing units" OR "DTU") AND ("Reservoirs" OR "Humans" OR "Vectors") AND Mexico.
- Search String 2: ("*Trypanosoma cruzi*" OR "Chagas disease") AND ("genetic characterization" OR "genotyping") AND ("Reservoirs" OR "Humans" OR "Vectors") AND Mexico.

### Data Extraction

Data extraction was performed independently by two reviewers using a standardized spreadsheet in the Statistical Package for the Social Sciences (SPSS). To ensure data homogeneity, findings from studies published prior to the current consensus nomenclature were reclassified based on the equivalencies described in the literature. Essential data required for the synthesis of results were collected from each included study, comprising:

- Study Identification: Article title and publication data.
- Georeferencing: Country, state (federal entity), municipality, and locality, as well as the sample collection date (when available).
- Sample Characteristics: Type of sample (e.g., blood, feces, intestinal content, or tissue), scientific name of the vector, reservoir, or host, and its classification according to the transmission cycle. To ensure accuracy and avoid interpretation bias, the assignment of epidemiological cycles (domestic, peridomestic, or sylvatic) was based strictly on the explicit description provided in the original articles. Samples lacking specific ecological context or explicit classification by the authors were categorized as “undefined cycle.”
- Genetic Results: Identification of DTUs and the presence of mixed infections, when reported.

Discrepancies in data extraction were resolved through consensus between both reviewers.

For the purposes of this review, a “record” refers to a unique biological sample successfully genotyped. In cases of mixed infections, the sample was counted as a single record to avoid overestimating host or vector totals. However, for the analysis of DTU frequency and distribution, each identified genotype was treated as an independent observation. This approach ensures an accurate representation of the parasite's genetic diversity.

## RESULTS

The systematic literature analysis revealed a dataset of 47 eligible studies (39 original research articles, 7 short communications, and 1 case report). These studies yielded a total of 1,337 individual records of *T. cruzi* DTUs reported from Mexico between 2006 and 2025. Of these records, 930 corresponded to triatomine vectors (order Hemiptera), 331 to non-human reservoir hosts (comprising eight taxonomic orders), and 51 to human cases. Additionally, 25 records lacked specific host or vector data (Supplementary Table S1).

### Methodological Approaches and Target Loci for *T. Cruzi* Genotyping

To determine the most prevalent strategies for *T. cruzi* genotyping, we analyzed the methodological approaches and molecular targets employed across the 47 reviewed studies. Regarding technical approaches (Supplementary Figure S2), sequencing and conventional PCR emerged as the predominant methodologies, implemented in 18 (38.3%) and 17 (36.2%) of the studies, respectively. Real-Time PCR was utilized in 6 studies (12.8%). Meanwhile, 3 studies (6.4%) based their lineage determination on pattern and profile analyses, including Random Amplified Polymorphic DNA (RAPD), microsatellites, and Multilocus Enzyme Electrophoresis (MLEE).

The Spliced Leader Intergenic Region (SL-IR) was the primary target for DTU assignment, used in 20 studies (42.6%) alone and in 12 (25.5%) with secondary markers Supplementary Table S2). Other independent markers or multilocus combinations (excluding SL-IR) were used in 7 studies (14.9%). Satellite DNA, kinetoplast DNA, and isoenzymes were also employed. Three articles (6.4%) did not specify genotyping methods but reported DTUs.

### Geographical Distribution of DTUs in Mexico

**FIGURE 1:**
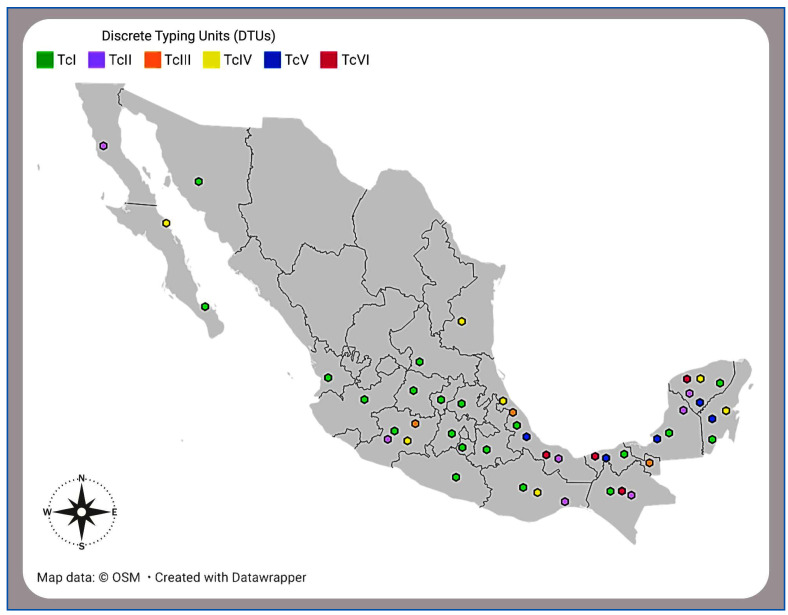
Geographic distribution of *Trypanosoma cruzi* discrete typing units (DTUs TcI-TcVI) reported in Mexico. This map illustrates the spatial distribution of the six main parasite lineages identified through a systematic review of the literature. Each record was individually analyzed to determine the specific circulating genotypes within each Mexican state. The visualization allows for the identification of areas with high genetic diversity and potential zones of genotype co-circulation. DTU: discrete typing unit; TcI-TcVI: *T. cruzi* genotypes.

Our analysis identified six of the seven main DTUs (TcI-TcVI) circulating in Mexico, with TcBat absent during the study period (Figure 1). This indicates multiple *T. cruzi* lineages across diverse ecological and epidemiological settings. At least one genetic variant has been documented in 22 of 32 Mexican states^17^ ^-^ ^23^ ^,^ ^25^ ^-^ ^31^ ^,^ ^36^ ^-^ ^68^, reflecting substantial but heterogeneous molecular surveillance. TcI was the most widespread lineage, present in 20 of the 22 analyzed states, confirming its high dispersal capacity. Veracruz reported all six DTUs (TcI-TcVI), making it the state with the most comprehensive genetic diversity record. Yucatan followed with five DTUs (all except TcIII). TcIII and TcVI had the most restricted distribution, each reported in only four states (Table 1). However, observed distribution patterns should be interpreted considering uneven sampling intensity across regions.

**TABLE 1:** Distribution of *Trypanosoma cruzi* discrete typing units (DTUs) according to Mexican state and epidemiological transmission cycle.

Mexican state	Epidemiological transmission cycle					
	Domestic	Peridomestic	Sylvatic	Captivity	Undefined	Reference
Baja California	TcII	-	-	-	-	^28^
Baja California Sur	TcI	-	TcI and TcIV	-	-	^31^ ^,^ ^36^
Campeche	TcI and TcII	TcI and TcII	TcI, TcII and TcV	-	TcI	^20^ ^,^ ^29^ ^,^ ^31^ ^,^ ^37^ ^-^ ^40^
Chiapas	TcI and TcII	-	TcI	-	TcI and TcVI	^17^ ^,^ ^22^ ^,^ ^26^ ^,^ ^41^ ^-^ ^43^
Estado de Mexico	-	-	-	-	TcI	^25^
Guanajuato	TcI	-	-	-	-	^44^ ^,^ ^45^
Guerrero	TcI	-	-	-	-	^44^
Hidalgo	-	TcI	-	-	TcI	^46^ ^,^ ^47^
Jalisco	TcI	TcI	TcI	-	TcI	^44^ ^,^ ^45^ ^,^ ^48^ ^-^ ^50^
Michoacan	TcI to TcIV	-	-	-	-	^23^ ^,^ ^31^
Morelos	TcI	TcI	TcI	-	TcI	^17^ ^,^ ^31^ ^,^ ^44^ ^,^ ^51^
Nayarit	-	-	-	-	TcI	^52^
Oaxaca	TcI, TcII, and TcIV	-	-	-	TcI	^19^ ^,^ ^22^ ^,^ ^31^ ^,^ ^44^ ^,^ ^45^ ^,^ ^53^
Puebla	-	-	-	-	TcI	^30^ ^,^ ^54^
Queretaro	TcI	-	-	-	TcI	^31^ ^,^ ^44^
Quintana Roo	TcI and TcIV	-	-	TcI and TcV	-	^20^ ^,^ ^29^
San Luis Potosí	TcI	-	-	-	-	^31^
Sonora	TcI	-	-	-	TcI	^44^ ^,^ ^55^
Tabasco	-	-	TcI, TcIII, TcV and TcVI	-	TcI	^28^ ^,^ ^52^
Tamaulipas	-	-	-	-	TcIV	^56^
Veracruz	TcI, TcII, and TcVI	-	TcI, TcII, TcIV, TcV, TcVI,	TcI and TcII	TcI to TcVI	^18^ ^,^ ^27^ ^,^ ^29^ ^,^ ^31^ ^,^ ^44^ ^,^ ^57^ ^-^ ^60^
Yucatan	TcI, TcII, TcIV, TcV, and TcVI	TcI	-	TcVI	TcI and TcII	^17^ ^,^ ^20^ ^,^ ^21^ ^,^ ^29^ ^,^ ^44^ ^,^ ^61^ ^-^ ^67^
Undefined	TcI	-	-	-	-	^68^

**Note: DTU:** discrete typing unit. **TcI-TcVI:** *Trypanosoma cruzi* genotypes. The symbol (-) indicates that no records were identified in the reviewed literature. The "Undefined" category refers to records where specific geographic or ecological transmission data were missing in the original source. The "Captivity" category denotes infected animals maintained in managed care facilities or Environmental Management Units (UMAs). Data for this distinct transmission dynamic derives from reference ^29^.

### Geographic and Taxonomic Heterogeneity of DTU Distribution

Stratification by state and host taxonomic order revealed significant ecological and regional heterogeneity. Southeastern Mexico, particularly Yucatan and Campeche, showed the greatest taxonomic breadth with DTUs across seven orders (Table 2). Chiapas followed with records spanning five orders (Chiroptera, Didelphimorphia, Hemiptera, Primates [human], and Rodentia). This broad multi-order distribution suggests complex transmission networks and sustained interaction among sylvatic, peridomestic, and domestic cycles. In these southeastern states, TcI and TcII were consistently detected across multiple host groups, indicating broad ecological plasticity and limited host restriction. The recurrent identification of identical DTUs in vectors, wild reservoirs, domestic animals, and humans supports active cross-cycle connectivity.

**TABLE 2:** Distribution of *Trypanosoma cruzi* discrete typing units (DTUs) according to Mexican state and taxonomic order.

Mexican state	Vectors	Human	Reservoirs order
Baja California	-	TcII	-
Baja California Sur	TcI and TcIV	-	-
Campeche	TcI and TcII	-	Artiodactyla (TcI and TcII), Carnivora (TcI and TcII), Chiroptera (TcI and TcII), Didelphimorphia (TcI), Primates (non-human) (TcV), Rodentia (TcI and TcII)
Chiapas	TcI, TcII and TcVI	TcI	Chiroptera (TcI), Didelphimorphia (TcI), Rodentia (TcI)
Estado de Mexico	TcI	-	-
Guanajuato	-	TcI	-
Guerrero	-	TcI	-
Hidalgo	TcI	-	-
Jalisco	TcI	TcI	Rodentia (TcI)
Michoacan	TcI to TcIV	-	-
Morelos	TcI	TcI	Rodentia (TcI)
Nayarit	-	-	Undefined (TcI)
Oaxaca	TcI, TcII, and TcIV	TcI	-
Puebla	TcI	TcI	Rodentia (TcI)
Queretaro	TcI	-	-
Quintana Roo	TcI and TcIV	-	Primates (non-human) (TcI and TcV)
San Luis Potosí	-	TcI	-
Sonora	TcI	TcI	-
Tabasco	-	-	Primates (non-human) (TcI, TcIII, TcV, and TcVI)
Tamaulipas	-	-	Carnivora (TcIV)
Veracruz	TcI to TcVI	TcI and TcII	Primates (non-human) (TcI and TcII), Strigiformes (TcII)
Yucatan	TcI, TcII, TcV and TcVI	TcI and TcII, TcV and TcVI	Artiodactyla (TcI and TcVI), Carnivora (TcI, TcII, TcIV to VI), Didelphimorphia (TcI and TcII), Primates (non-human) (TcVI), Rodentia (TcI)

**Note: DTU:** discrete typing unit; **TcI-TcVI:** *Trypanosoma cruzi* genotypes. The symbol (-) indicates that no records were identified in the literature. The term 'Undefined' refers to records where specific geographic or ecological transmission data were missing in the original source.

TcV and TcVI were identified in Yucatan in triatomines, domestic carnivores (*Canis lupus familiaris* and *Felis catus*), and humans. Their multi-host detection in southeastern Mexico may reflect historical introduction with subsequent local establishment or improved molecular resolution. While the concentration of DTU-host diversity in the southeast aligns with high regional biodiversity and favorable ecological conditions, it also coincides with intensified research; thus, apparent genetic complexity should be interpreted considering heterogeneous sampling effort.

Central and northern states showed more restricted and host-specific reporting. In Guanajuato, Guerrero, and San Luis Potosi, molecular records were limited to human cases (all TcI), while in the State of Mexico, Hidalgo, Michoacan, and Queretaro, genotyping focused almost entirely on vectors. These asymmetric patterns likely reflect differences in surveillance strategies (clinical versus entomological) rather than a true absence of DTU diversity. Veracruz reported infection in birds (Strigiformes, TcII), though this requires cautious interpretation due to limited avian reservoir competence. In Tamaulipas, the only record involved Carnivora (TcIV), highlighting minimal sampling. Overall, the data position southeastern Mexico as a potential hotspot of *T. cruzi* genetic diversity and multi-order circulation, while central and northern regions appear under-characterized in terms of integrated eco-epidemiological surveillance.

### Epidemiological Transmission Cycles

**FIGURE 2:**
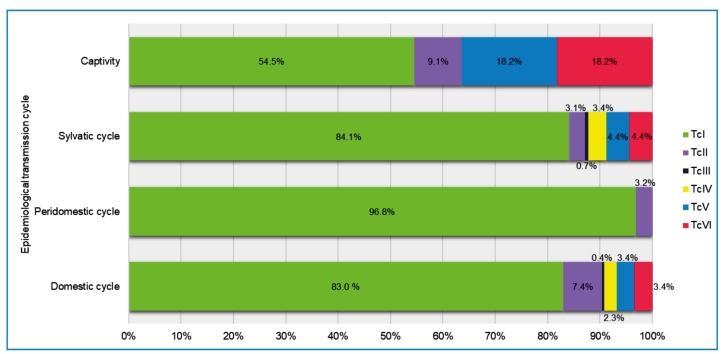
Distribution of *Trypanosoma cruzi* discrete typing units (DTUs) across different epidemiological transmission cycles in Mexico. Values represent the relative frequency of each identified genotype. The sample size (n) reflects the total number of DTU identifications per cycle: Domestic (n=470), Peridomestic (n=349), Sylvatic (n=295), and Captivity (n=11). These totals account for mixed infections by counting each detected genotype as an independent identification. The "Captivity" category denotes infected animals maintained in managed care facilities or Environmental Management Units (UMAs). Data for this distinct transmission dynamic are derived from reference 29. Records were excluded if the transmission cycle was undefined.

This geographical heterogeneity at the state level was mirrored by variation across epidemiological transmission cycles. Records showed more studies in the domestic cycle (470) compared to the sylvatic cycle (295), reflecting either intensified surveillance in human-associated settings or increased research focus on domiciliated vectors and reservoirs. Despite this sampling imbalance, all six reported DTUs (TcI-TcVI) were detected in both domestic and sylvatic contexts, indicating no lineage is strictly confined to a single transmission cycle in Mexico. Across all ecological scenarios, TcI consistently emerged as the predominant lineage (Figure 2), reinforcing its broad ecological plasticity and capacity to circulate across multiple host assemblages and environmental settings.

### Vectors

**FIGURE 3:**
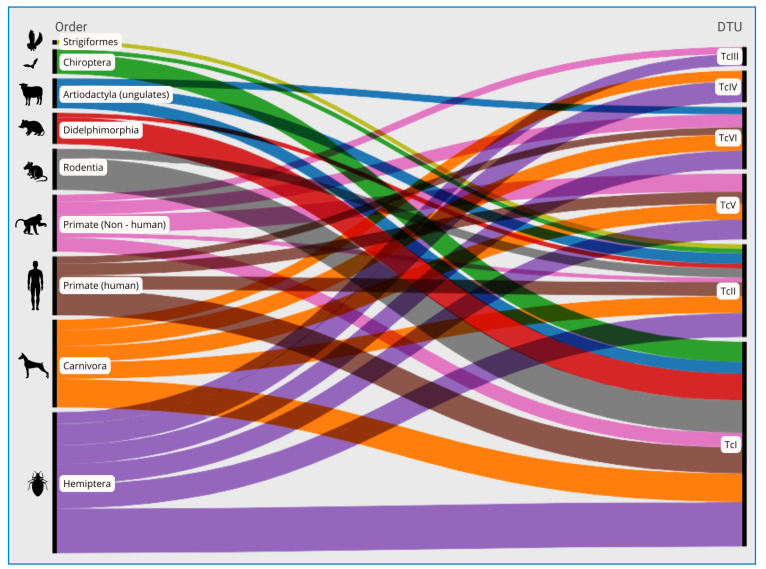
Bipartite network of *Trypanosoma cruzi* discrete typing units (DTUs) across taxonomic orders in Mexico. The diagram illustrates the ecological interactions between nine taxonomic orders (left) and six *T. cruzi* DTUs (right). The width of the flows represents the frequency of records for each interaction. To facilitate the visualization of both dominant and rare interactions, flow widths were scaled using a log10 (n+1) transformation of the absolute counts. Icons indicate the respective taxonomic groups, and colors differentiate the DTUs. This network highlights the high connectivity of vectors (Hemiptera), which bridge all six DTUs. Furthermore, it demonstrates the broad host range of TcI across diverse mammalian orders, contrasting with the more restricted connectivity observed for TcIII and TcIV. The visualization was created using RAWGraphs software.

Among the nine taxonomic orders analyzed, Hemiptera accounted for the greatest number of DTU records. As illustrated in the network analysis (Figure 3), triatomine vectors act as the central epidemiological node, encompassing and bridging all six reported *T. cruzi* DTUs (TcI-TcVI). Within this order, the genus *Triatoma* contributed the highest number of genotyped records (n=897), representing 67% of the total records analyzed in this study (Supplementary Figure S3). These findings likely reflect the high species richness and wide distribution of the genus in Mexico, though this proportional representation may also be influenced by the heterogeneity of sampling efforts across the analyzed studies. Within this genus, the species *Triatoma dimidiata* and *T. longipennis* stood out for having the highest number of records. *T. longipennis* was predominantly associated with TcI in peridomestic and sylvatic cycles; however, non-TcI variants (without specific typing between TcII-TcVI) were also reported in the sylvatic cycle. *T. dimidiata* exhibited the highest genetic diversity, with reports of all six DTUs (TcI-TcVI). This identifies it as the primary bridge vector in Mexico, capable of mobilizing *T. cruzi* genetic diversity between sylvatic and human environments. This species occurred in the domestic cycle (all DTUs except TcIII), the peridomestic cycle (TcI and TcII), and the sylvatic cycle (TcI, TcII, TcIV, and TcVI). The TcIII DTU was linked to this species only in records where the epidemiological cycle remained unspecified.

Other significant genera included *Dipetalogaster maxima*, associated with TcI in the domestic cycle and TcI and TcIV in the sylvatic cycle. *Panstrongylus rufotuberculatus* was detected exclusively with TcI in both sylvatic and domestic environments. These more restricted lineage associations may reflect either ecological specificity or limited molecular sampling.

### Hosts and Reservoirs

Human isolates yielded four genetic variants: TcI, TcII, TcV, and TcVI. TcI exhibited the broadest geographic distribution in humans, with records spanning eleven states (Chiapas, Guanajuato, Guerrero, Jalisco, Morelos, Oaxaca, Puebla, San Luis Potosi, Sonora, Veracruz, and Yucatan), reinforcing its established epidemiological predominance in Mexico. In contrast, TcII appeared in humans only in Baja California, Veracruz, and Yucatan, while TcV and TcVI remained restricted to Yucatan.

Beyond human infections, TcI and TcII were detected across most of the evaluated taxonomic orders. The interconnectivity map clearly demonstrates this broad host range and ecological versatility **(Figure 3)**, visually underscoring how these variants link to diverse mammalian groups. This widespread distribution contrasts with the more limited detection of TcIII and TcIV. As depicted in the network, TcIII connectivity was exclusively restricted to hemipterans and non-human primates. Similarly, TcIV exhibited a highly focal network, linking exclusively to hemipterans and domestic carnivores. However, this apparent host restriction should be interpreted cautiously, as it may be influenced by uneven sampling intensity and differential genotyping resolution among studies.

Collectively, these patterns highlight TcI as the dominant lineage across both human and non-human hosts in Mexico, while non-TcI DTUs appear more geographically and taxonomically focal. The presence of multiple DTUs in human infections (particularly TcII, TcV, and TcVI) underscores the need for continued molecular surveillance, given potential lineage-associated differences in clinical presentation and transmission dynamics reported in other endemic regions.

## DISCUSSION

This study synthesizes the geographical occurrence of *T. cruzi* DTUs based on published molecular records; it is not a measure of prevalence. The distribution of 1,337 analyzed records reflects heterogeneous sampling, shaped by historical research priorities in established endemic regions rather than solely by biological determinants. Thus, distinguishing between a lack of molecular evidence and biological exclusion of the pathogen is critical. These gaps represent a deficiency in characterization, not the true absence of *T. cruzi*, underscoring the necessity of expanding surveillance to understudied regions to capture the parasite's full genetic diversity.

The concentration of reports in central and southern Mexico aligns with the historical view of CD as a pathology linked to socioeconomic vulnerability and tropical or temperate climates, conditions favoring triatomine vector proliferation and diversity^3^ ^,^ ^4^ ^,^ ^6^. Nonetheless, isolated data from northern Mexico suggest the disease is not exclusive to southern regions but has been understudied there due to geographic distribution dogma. Competent vectors are established in these latitudes; therefore, without timely surveillance and control, autochthonous cases may continue to go undetected.

Our findings confirm that TcI is the lineage with the widest distribution and highest frequency in Mexico, appearing in most analyzed states. This aligns with continental reviews positioning TcI as predominant in North and Central America^14^ ^,^ ^15^. Its success is likely due to high genetic variability, allowing it to circulate effectively in domestic and sylvatic cycles, infecting a broad range of taxonomic orders^16^. Furthermore, the dichotomy between domestic and sylvatic cycles is breaking down. Our data show DTUs traditionally considered “sylvatic” (TcIII, TcIV) are present in domestic environments, while “domestic” lineages (TcII, TcV, TcVI) occur in the wild^12^ ^,^ ^15^. This exchange is facilitated by synanthropic animals (opossums, rats) and domestic animals (dogs, cats) acting as epidemiological bridges^51^. This bidirectional flow characterizes an integrated system where wild reservoirs sustain DTU diversity in human settlements, ensuring dynamic domestic transmission. Therefore, control measures must account for ecological connectivity between sylvatic and domestic environments. We must transition from exclusive indoor residual spraying to integrated management strategies, including eliminating synanthropic refuges and improving housing materials to reduce colonization by bridge vectors.

Among domestic reservoirs, dogs are critically relevant as significant peridomestic reservoirs and sentinels for *T. cruzi* transmission^69^. Our findings reinforce this, as dogs exhibited greater genetic diversity than humans, harboring five of the seven DTUs (TcI, TcII, and TcIV-TcVI). Domestic carnivores in our bipartite network displayed diversity spanning nearly all lineages, including TcIV. This demonstrates these animals are not only in constant contact with vectors from sylvatic foci but also serve as the primary link maintaining sylvatic diversity within human environments. Therefore, utilizing dogs as sentinels could strengthen public health surveillance. Monitoring sylvatic DTUs in canine populations provides an early warning system for human spillover, especially in areas with high ecological connectivity. Tracking these genotypes in dogs allows authorities to anticipate and mitigate risks before human cases emerge.

The high frequency of vector records (67%) underscores their role as the primary epidemiological bridge between transmission cycles. This also highlights significant heterogeneity in sampling efforts across the analyzed literature, suggesting molecular research in Mexico has historically prioritized entomological surveillance. Consequently, human infections and non-human reservoirs remain comparatively less characterized. The prominence of *T. dimidiata* with the most records and DTU diversity can be explained by its remarkable capacity for domiciliation and extensive geographic range in southeastern Mexico^20^ ^,^ ^26^. Its ubiquity in human settlements makes it the primary driver of current epidemiological reports.

An interesting finding is the record of the TcII genotype in Strigiformes. Although birds have historically been considered refractory to *T. cruzi* infection, recent evidence documents natural infections challenging this paradigm^60^. This finding expands Mexico's known vertebrate host pool and suggests birds may play a role in maintaining genetic diversity within transmission cycles, rather than being just blood sources for vectors. Conversely, the total absence of TcBat reports is notable. This information void can be attributed to molecular studies in Mexico not prioritizing targeted sampling of bats, as only 2.1% (29 of 1,337) of our total records come from this order. Given that TcBat has been identified in neighboring Central and South American countries^70^ ^,^ ^71^, designing specific detection protocols for this lineage in Mexican chiropterans is imperative to confirm or rule out its presence. Additionally, the limited diversity in human samples (TcI, TcII, TcV, TcVI) contrasts with the high variability in domestic dogs, which harbor nearly the full spectrum of DTUs circulating in Mexico. This discrepancy likely results from diagnostic timing^8^ ^,^ ^68^. While the acute phase facilitates molecular typing, these cases are rarely detected. Most human records stem from the chronic phase, where extremely low parasite loads significantly hinder characterization.

Our findings confirm that DTUs traditionally associated with sylvatic environments, such as TcIII and TcIV, are established in domestic settings. Their presence in dogs and vectors provides evidence of constant spillover from wild reservoirs. Consequently, human populations are likely to be equally exposed to this diversity. The apparent absence of these sylvatic DTUs in humans likely reflects technical constraints rather than a lack of biological exposure. Therefore, high-sensitivity tools are essential to detect low-load chronic infections. Genotyping is fundamental, as DTU diversity could potentially influence drug efficacy and clinical presentation^33^.

A critical aspect influencing the understanding of this genotypic diversity is the marked heterogeneity in methodological strategies employed over time. Our analysis revealed that sequencing and conventional PCR are predominant. Furthermore, the spliced-leader intergenic region (SL-IR) is the primary molecular anchor for DTU assignment, utilized as a single target in a considerable proportion of studies. Although SL-IR is a highly informative and validated marker, lack of standardization and reliance on a single marker limit typing precision. This methodological variability underscores the need to transition toward multilocus schemes or higher-sensitivity genomic tools for direct, standardized data comparability across different regions and epidemiological scenarios.

This review has methodological and conceptual limitations. First, reliance on existing literature introduces inherent biases related to asymmetrical sampling efforts focused on established endemic regions. Second, there is a risk of DTU misclassification due to variability in genotyping approaches and single markers. Additionally, characterizing mixed infections is a significant technical challenge. Conventional molecular techniques, by favoring the amplification of the predominant genotype (typically TcI), tend to underestimate other DTUs. This limitation could mask the true prevalence of minority strains and the complexity of coinfections within transmission cycles.

Despite these technical limitations, this review provides a scientific basis for precision-based surveillance in biodiverse Mexico. By synthesizing *T. cruzi* DTU distribution and highlighting knowledge gaps, it addresses the need to understand the impact of genetic diversity on human and animal health.

## CONCLUSION

*T. cruzi* genetic diversity in Mexico is broader and more ecologically interconnected than traditionally assumed. Six DTUs (TcI-TcVI) have been documented across multiple states, host taxa, and transmission cycles, though their apparent distribution is influenced by uneven molecular surveillance. TcI dominates the national landscape, reflecting its ecological plasticity and capacity to circulate across sylvatic and domestic environments. The detection of non-TcI DTUs in southeastern Mexico reveals a more complex genetic structure than historically recognized for North America, including lineages like TcV and TcVI, which are rarely recorded in wild environments. The dissolution of a strict domestic-sylvatic dichotomy further highlights the permeability of transmission cycles, mediated by generalist vectors and bridge hosts such as dogs.

At the same time, regional asymmetry in published records exposes significant surveillance gaps, especially in northern Mexico and in under-sampled host groups such as bats. The absence of reports should not be interpreted as a biological absence but as a reflection of limited molecular characterization. Advancing toward precision-based epidemiological surveillance in Mexico will require high-sensitivity genotyping in humans, systematic vector-reservoir integration, and targeted studies in ecologically diverse but understudied regions. Mapping the true extent of DTU circulation is essential for understanding transmission dynamics and anticipating potential clinical and epidemiological implications of different genotypes in Mexico.

## Data Availability

All relevant data are within the manuscript. Raw datasets analyzed during the current study are available from the corresponding author on reasonable request.
