# Supplementary material for: Genetic diversity and geographical distribution of Trypanosoma cruzi DTUs in Mexico: A Systematic Review
Source: Rev Soc Bras Med Trop. 2026 Aug 3;59:e0126-2026. doi: 10.1590/0037-8682-0126-2026 (PMC13432801; doi:10.1590/0037-8682-0126-2026)
Supplement: Supplementary Figure S1 [file 1678-9849-rsbmt-59-e0126-2026-md1.pdf]

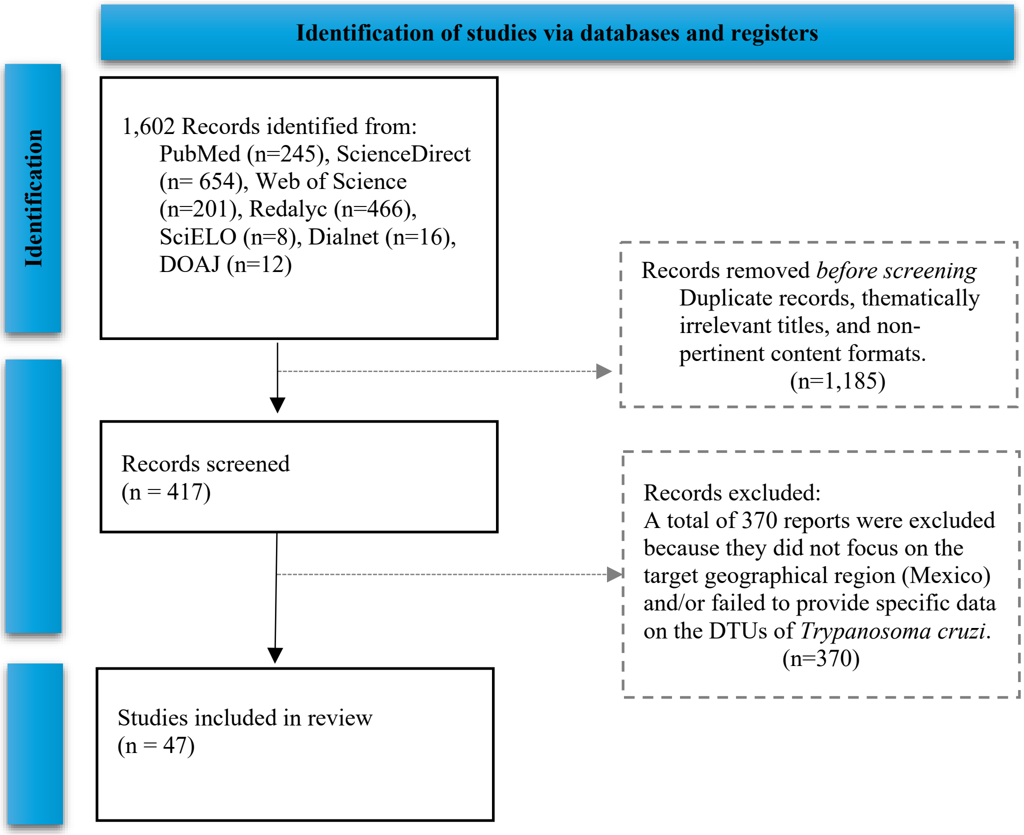

FIGURE S1: Systematic workflow for the selection of articles for inclusion in the review of *Trypanosoma cruzi* discrete typing units (DTUs) distribution in Mexico. This flow diagram details the literature search and screening process. An initial search across seven academic databases yielded 1,602 records. During the identification phase, 1,185 records were removed prior to screening. Reasons for removal included duplicate records, irrelevant titles, and non-pertinent content formats. Subsequently, 417 records underwent a detailed screening process. Of these, 370 reports were excluded. Exclusions were made if the studies did not focus on the target geographical region (Mexico) or if they failed to provide specific data on *T. cruzi* DTUs. Ultimately, 47 studies met all inclusion criteria and were included in the final review.
