# Supplementary material for: Genetic diversity and geographical distribution of Trypanosoma cruzi DTUs in Mexico: A Systematic Review
Source: Rev Soc Bras Med Trop. 2026 Aug 3;59:e0126-2026. doi: 10.1590/0037-8682-0126-2026 (PMC13432801; doi:10.1590/0037-8682-0126-2026)
Supplement: Supplementary Figure S2 [file 1678-9849-rsbmt-59-e0126-2026-md2.pdf]

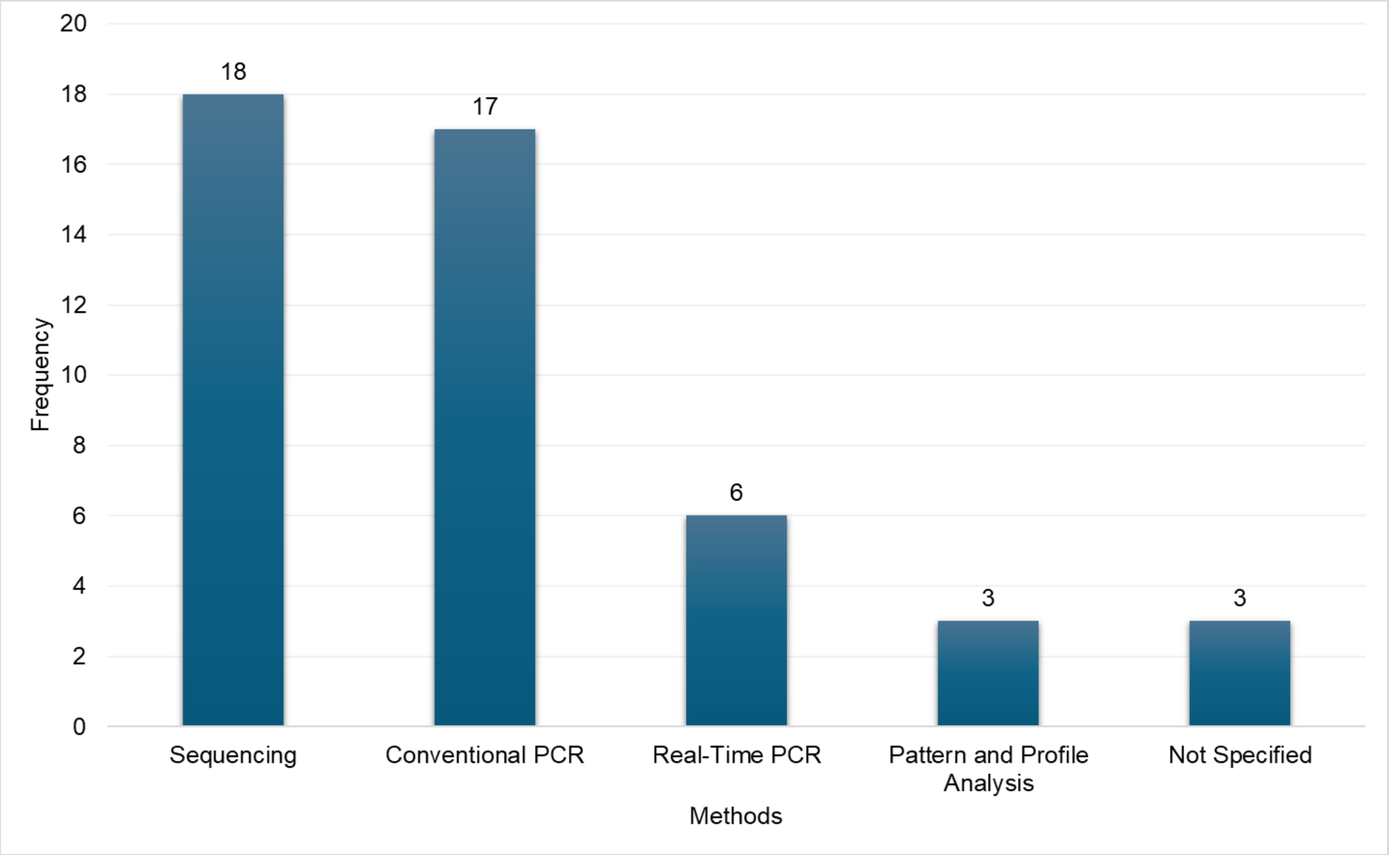

FIGURE S2: Frequency of methodological approaches used for *Trypanosoma cruzi* genotyping. The bar chart illustrates the distribution of the main molecular methods employed across the included studies for the identification of discrete typing units (DTUs) (N = 47). Sequencing and conventional PCR stand out as the predominant methodologies in the analyzed literature. For the frequency analysis, studies reporting mixed methodological approaches were categorized based on the technique with the highest sensitivity and analytical resolution used for the final genotype confirmation. It should be noted that the "Pattern and Profile Analysis" category groups three articles that based their typing on the evaluation of polymorphic band patterns or electrophoretic profiles, specifically: Random Amplified Polymorphic DNA (RAPD), microsatellite analysis, and Multilocus Enzyme Electrophoresis (MLEE). Finally, a "Not Specified" category was included for studies that did not detail the exact methodology but did report the discrete typing units (DTUs) found in their results.
