# Supplementary material for: Genetic diversity and geographical distribution of Trypanosoma cruzi DTUs in Mexico: A Systematic Review
Source: Rev Soc Bras Med Trop. 2026 Aug 3;59:e0126-2026. doi: 10.1590/0037-8682-0126-2026 (PMC13432801; doi:10.1590/0037-8682-0126-2026)
Supplement: Supplementary Figure S3 [file 1678-9849-rsbmt-59-e0126-2026-md3.pdf]

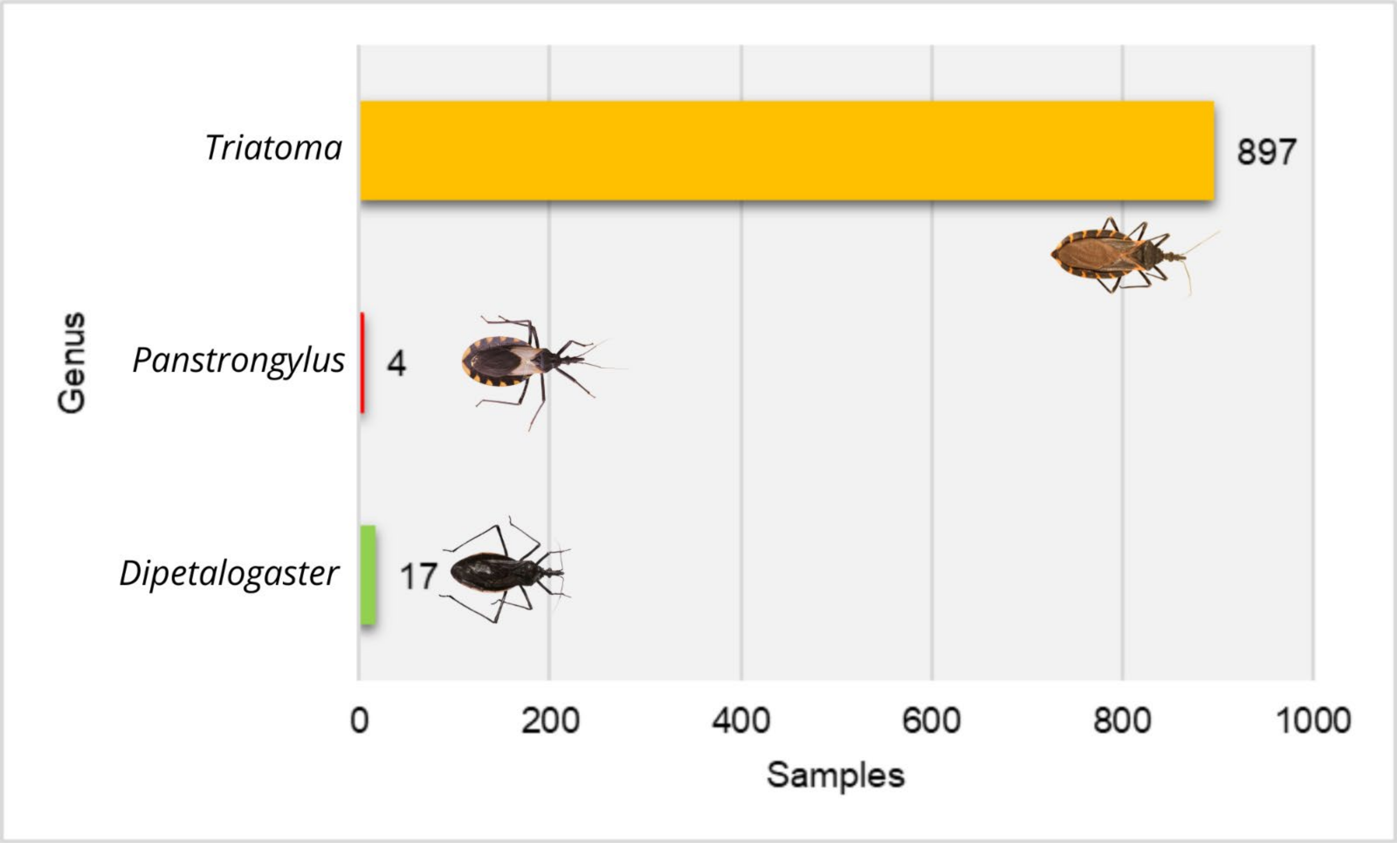

FIGURE S3: Frequency of records by triatomine genus associated with the identification of *Trypanosoma cruzi* discrete typing units (DTUs) in Mexico. This graph illustrates the distribution of the analyzed triatomine vectors (N = 918). The samples are categorized into three distinct genera. *Triatoma* emerges as the predominant genus, accounting for 897 records. *Dipetalogaster* and *Panstrongylus* represent 17 and 4 records, respectively. The data originates from an initial pool of 930 vector records. However, 12 nymphs were excluded from this final analysis. This exclusion was necessary due to a lack of specific taxonomic identification at the species level.
