# Supplementary material for: Genetic diversity and geographical distribution of Trypanosoma cruzi DTUs in Mexico: A Systematic Review
Source: Rev Soc Bras Med Trop. 2026 Aug 3;59:e0126-2026. doi: 10.1590/0037-8682-0126-2026 (PMC13432801; doi:10.1590/0037-8682-0126-2026)
Supplement: Supplementary Table S1 [file 1678-9849-rsbmt-59-e0126-2026-md4.pdf]

TABLE S1: Total frequency of genotyped *Trypanosoma cruzi* records by taxonomic order in Mexico (2006–2025).

Taxonomic Order	Number of Records (n)	Percentage (
Hemiptera (Vectors)	930	69.56%
Rodentia	132	9.87%
Carnivora	77	5.76%
Didelphimorphia	57	4.26%
Primates (Human)	51	3.82%
Chiroptera	29	2.17%
Primates (Non-humar	27	2.02%
Artiodactyla	8	0.60%
Strigiformes	1	0.07%
Undefined / No data	25	1.87%
Total (N)	1,337	100%

Note: The total (N = 1,337) includes data from triatomine vectors, human cases, and non-human reservoirs reported in Mexico between 2006 and 2025. The category "Undefined / No data" refers to records where specific host or vector information was not provided in the original source. N: total number of records; n: number of records per category; %: relative frequency.
