# Supplementary material for: Genetic diversity and geographical distribution of Trypanosoma cruzi DTUs in Mexico: A Systematic Review
Source: Rev Soc Bras Med Trop. 2026 Aug 3;59:e0126-2026. doi: 10.1590/0037-8682-0126-2026 (PMC13432801; doi:10.1590/0037-8682-0126-2026)
Supplement: Supplementary Table S2 [file 1678-9849-rsbmt-59-e0126-2026-md5.pdf]

TABLE S2: Genotyping algorithms and target loci used for *Trypanosoma cruzi* characterization.

Genotyping algorithms and target loci	Number of studies
SL-IR	20
SL-IR + 24Sα/18S rDNA/A10/ND1/COII/microsatellites	12
Other markers[a]	7
Sat-DNA/kDNA	4
Isoenzyme[b]	1
Not specified[c]	3
Total	47

Note. The data represents the genotyping strategies identified across the 47 reviewed articles.

[a] This category groups studies using markers either alone or in combination, independently of the SL-IR target. Loci include: 24Sα/18S rDNA, GPI, COII, ND1, CytB, C-5 desaturase, SSU rDNA, gGAPDH, DHFR-TS, TRY2, TcCLB.508213.20, and Rb19.

[b] This category encompasses the 20 specific isoenzymes standardly utilized for *Trypanosoma cruzi* characterization; for a complete detailed list of these markers, see reference 30.

[c] Studies in this category reported the final discrete typing units (DTUs) identification but did not explicitly state the molecular marker or genotyping methodology utilized to achieve it.
